# Boron-Based Neutron Scintillator Screens for Neutron Imaging

**DOI:** 10.3390/jimaging6110124

**Published:** 2020-11-19

**Authors:** William Chuirazzi, Aaron Craft, Burkhard Schillinger, Steven Cool, Alessandro Tengattini

**Affiliations:** 1 Advanced Post-Irradiation Examination Department, Idaho National Laboratory, Idaho Falls, ID 83401, USA; aaron.craft@inl.gov; 2 Heinz Maier-Leibnitz Zentrum (FRM II) and Faculty for Physics E21, Heinz Maier-Leibnitz Zentrum (FRM II), Technische Universität München, 85748 Garching, Germany; burkhard.schillinger@frm2.tum.de; 3 DMI/Reading Imaging, Reading, MA 01867, USA; stever70@aol.com; 4 Le Centre National de la Recherche Scientifique (CNRS), Université Grenoble Alpes, Grenoble INP, 3SR, 38000 Grenoble, France; alessandro.tengattini@3sr-grenoble.fr; 5 Institut Laue-Langevin (ILL), 71 Avenue des Martyrs, 38000 Grenoble, France

**Keywords:** neutron imaging, neutron radiography, digital imaging, neutron scintillator, scintillator screen, boron scintillator, scintillator characterization, scintillator detection efficiency, epithermal neutron imaging, neutron sensor

## Abstract

In digital neutron imaging, the neutron scintillator screen is a limiting factor of spatial resolution and neutron capture efficiency and must be improved to enhance the capabilities of digital neutron imaging systems. Commonly used neutron scintillators are based on ^6^LiF and gadolinium oxysulfide neutron converters. This work explores boron-based neutron scintillators because ^10^B has a neutron absorption cross-section four times greater than ^6^Li, less energetic daughter products than Gd and ^6^Li, and lower γ-ray sensitivity than Gd. These factors all suggest that, although borated neutron scintillators may not produce as much light as ^6^Li-based screens, they may offer improved neutron statistics and spatial resolution. This work conducts a parametric study to determine the effects of various boron neutron converters, scintillator and converter particle sizes, converter-to-scintillator mix ratio, substrate materials, and sensor construction on image quality. The best performing boron-based scintillator screens demonstrated an improvement in neutron detection efficiency when compared with a common ^6^LiF/ZnS scintillator, with a 125% increase in thermal neutron detection efficiency and 67% increase in epithermal neutron detection efficiency. The spatial resolution of high-resolution borated scintillators was measured, and the neutron tomography of a test object was successfully performed using some of the boron-based screens that exhibited the highest spatial resolution. For some applications, boron-based scintillators can be utilized to increase the performance of a digital neutron imaging system by reducing acquisition times and improving neutron statistics.

## 1. Introduction

### 1.1. Background

Neutron imaging is a nondestructive examination technique that measures neutron transmission to examine a sample’s internal structure. This technique has been used in a variety of studies, including those of nuclear fuels and materials [1,2,3,4,5,6], cultural heritage objects [7,8,9], fuel cells [10,11,12,13,14], turbine blades [15,16,17], and many others. Digital neutron imaging techniques are now an essential capability for any state-of-the-art neutron imaging facility [18]. The most common digital neutron imaging system consists of a digital camera optically coupled to a neutron-sensitive scintillator screen. 

A critical component of digital neutron imaging systems is the neutron scintillator, because the scintillator dictates the neutron capture efficiency and is a limiting factor in spatial resolution. Common scintillator screens for thermal and cold neutron imaging utilize either a ^6^Li(n,α)^3^H reaction due to its large neutron absorption cross-section and relatively large reaction energy, or a Gd(n,γ + IC_e−_) reaction because of gadolinium’s high neutron absorption cross-section [19,20,21,22,23]. Although these scintillator screens are the state of the art in the digital neutron imaging community, the continual improvement of neutron scintillators are necessary to meet the ever-higher user demands for increased spatial resolution to examine smaller samples and increased detection efficiency to decrease measurement time.

**Figure 1:**
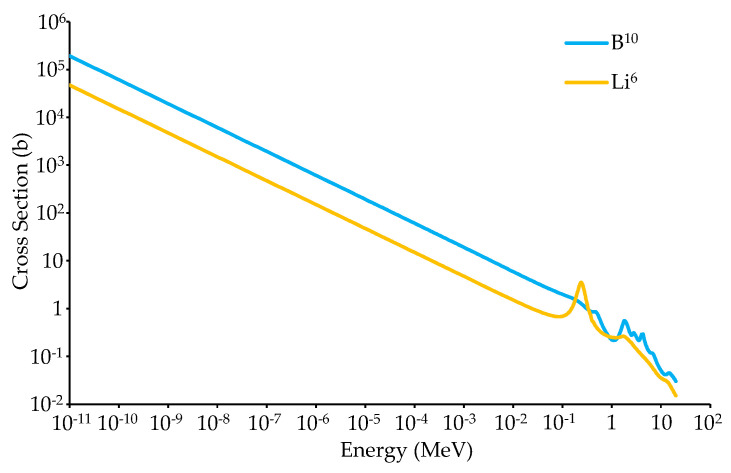
Microscopic absorption cross-section of ^10^B and ^6^Li from the Evaluated Nuclear Data File (ENDF/B-VIII) cross-section libraries.

Boron-based screens offer a promising alternative to existing ^6^Li- and Gd-based neutron scintillator screens for a number of reasons. First, the thermal neutron cross-section of ^10^B (3840 b) is approximately four times greater than that of ^6^Li (980 b), as shown in Figure 1, which should increase the neutron detection efficiency for a comparable ^6^Li-based screen. Second, the daughter products created by neutron absorption of ^10^B have less total energy (2.31 MeV or 2.79 MeV) than those of ^6^Li (4.78 MeV) and Gd (7.937 MeV or 8.536 MeV), and the lower energy from a ^10^B(n,α)^7^Li reaction is distributed on heavier daughter products compared to a ^6^Li(n,α)^3^H reaction. The lower energy and larger size of the daughter products reduces the range the daughter products will travel, a fundamental limiting factor of a screen’s spatial resolution, potentially enabling borated scintillator screens to exhibit higher spatial resolution compared to the screens created with ^6^Li or Gd. Additionally, ^10^B has a lower γ-ray sensitivity than gadolinium due to its lower atomic number. Scintillator screens with a boron converter can theoretically be made much thinner than current ^6^Li- and Gd-based screens, while exhibiting superior neutron detection efficiency to ^6^Li scintillator screens, improved γ-ray insensitivity over Gd scintillator screens, and improved spatial resolution. The combination of ^10^B’s improved neutron detection efficiency, spatial resolution, and γ-ray insensitivity could potentially provide a high-performance neutron scintillator.

Boron-based neutron scintillators for neutron imaging applications were conceived as early as the 1950s [24,25] and their superior neutron detection efficiency has been documented [26]. Glass scintillators [27,28], scintillators with a mixture of neutron absorbing materials [29,30], and plastic scintillators [31,32,33,34] have all utilized boron for neutron detection in different contexts. Despite their usefulness in other applications, previous studies have generally concluded that boron-based scintillator screens’ poor light output prevented them from becoming a viable scintillator for neutron imaging [35,36,37]. However, Nakamura et al.’s work [38] suggests smaller boron particles may increase light output performance. More recent efforts by other colleagues in the neutron imaging field have recently explored using ^10^B/CsI:Tl scintillator screens for neutron imaging [39].

The working hypothesis of this study is that the low probability of ^10^B’s daughter products to escape from the boron-containing particle and arrive as a scintillating particle is the likely cause of the low light output observed in previous investigations of boron-based neutron scintillator screens. This work aims to use smaller boron converter particles to enhance light output. The goal of this investigation is to benefit the broader neutron imaging community by exploring boron-based scintillator screens that may deliver improved neutron detection efficiency, light output, and potentially spatial resolution.

### 1.2. Theory of Boron-Based Scintillator Screens

The range of daughter products created when a neutron interacts with a neutron converter is one of the limiting factors of a scintillator screen’s spatial resolution. When a thermal neutron (0.025 eV) is absorbed by ^10^B, a ^10^B(n,α)^7^Li reaction occurs with a 94% branching ratio of producing a ^7^Li particle in an excited state (Equation (1)) and a 6% branching ratio of producing a ^7^Li in the ground state (Equation (2)), with an average reaction energy of 2.34 MeV. The daughter products of ^10^B have less energy than those created by thermal neutron interactions with ^6^Li (Equation (3)) and Gd (Equations (4) and (5)), causing them to deposit energy more locally to the interaction site and improve the theoretical spatial resolution limit. Additionally, ^10^B’s daughter products are more massive and deposit their energy over a shorter range than those of ^6^Li:

(1) B510+n01→L37i*(0.84 MeV)+α24(1.47 MeV)

(2) B510+n01→L37i(1.01 MeV)+α24(1.78 MeV)

(3) L36i+n01→H13(2.73 MeV)+α24(2.05 MeV)

(4) G64157d+n01→G64158d*→G64158d+γ rays+ICe−;Q=7.937 MeV

(5) G64155d+n01→G64156d*→G64156d+γ rays+ICe−;Q=8.536 MeV

Natural gadolinium, which consists of seven stable isotopes, has a total thermal neutron cross-section of 48,800 barns. Two isotopes that have high thermal neutron capture cross-sections and a relatively high natural abundance are ^155^Gd (60,900 b, 14.8% abundance) and ^157^Gd (254,000 b, 15.7% abundance) [40]. As shown in Equations (4) and (5), gadolinium neutron absorption reactions produce prompt γ-rays and internal conversion electrons (IC_e−_), which also produce secondary characteristic X-rays and Auger electrons, including Coster–Kronig electrons. The γ-rays represent 99% of the Q-value energy [41], but the emitted electrons deposit their energy more locally than photons. Thus, the response of a gadolinium oxysulfide scintillator (e.g., GOS, Gd_2_O_2_S, informally known as Gadox) to thermal neutron capture is a combination of the effects of emitted photons and electrons. Different GOS scintillators (such as GOS:Tb, GOS:Pr, GOS:Eu) have different properties, such as peak light emission, photons/MeV, and decay time. These properties can be matched to the specific application requirements to maximize performance. Thermal neutron capture by ^157^Gd, for example, releases an average of 3.288 photons including prompt and secondary γ-rays and X-rays with energies ranging from 10s of eV up to several MeV and a mean energy of 2.394 MeV [42,43]. This same reaction also releases IC_e−_ with energies ranging from 29 keV to 6.9 MeV, with the most intense discrete IC_e−_ emissions of 29 and 71 keV [40,43,44]. The range of a 71 keV IC_e−_ in gadolinium is approximately 20 µm [45]. Goorley and Nikjoo have compiled a more complete table of gadolinium spectral photon emissions [43]. 

Less energetic daughter products help improve spatial resolution because they have a shorter range in the surrounding material. The probability that the daughter products will escape the boron converter particles and reach the scintillator particles decreases with increasing converter particle size. When the converter particles themselves prevent daughter products from reaching the scintillator material and producing photons, the light output of the scintillator screen is reduced despite any apparent improvement in the detection efficiency from using ^10^B compared to ^6^Li. Thus, the converter particle size could explain the poor light output reported by previous studies on borated scintillator screens.

**Figure 2:**
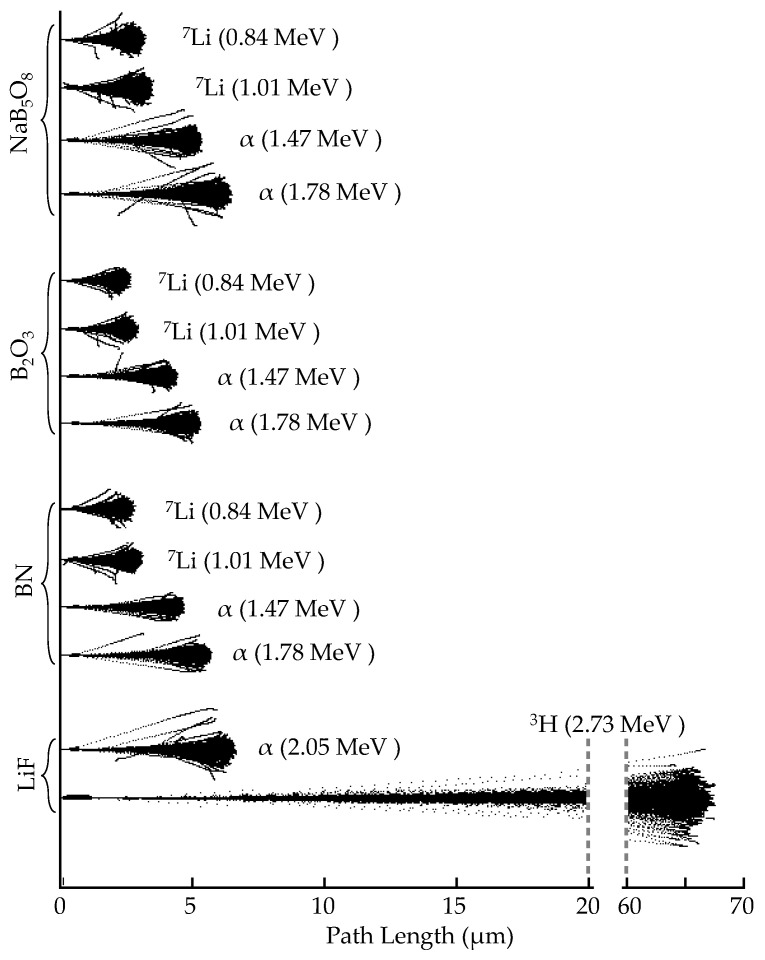
Transmission of Ions in Matter (TRIM) simulated daughter product path length in various converter materials. The ^10^B(n,α)^7^Li daughter products deposit their energy more locally than the ^6^Li(n,α)^3^H daughter products. This demonstrates that ^10^B-based scintillators offer an improved theoretical spatial resolution limit compared to ^6^Li-based screens.

A Transmission of Ions in Matter (TRIM) simulation [46] calculated the ranges of the daughter products from the neutron absorption reactions of interest in the boron and lithium-based scintillator screens. TRIM does not account for isotopic information, so natural elemental abundances were used in the calculation. The range of internal conversion electrons in GOS scintillator screens was calculated using the Penetration and Energy Loss of Positrons and Electrons (PENELOPE) software (Version) [47]. Table 1 lists the daughter particles in the converter material, the converter material densities used for the simulation, and the resulting daughter product ranges, which are also displayed in Figure 2.

These simulations show ^6^Li daughter products traveling through LiF, as well as ^10^B daughter products traveling through a layer of B_2_O_3_, NaB_5_O_8_, or BN. The densities of NaB_5_O_8_ and Na^10^B_5_O_8_ were essential to analyzing the imaging results, but the density of anhydrous NaB_5_O_8_ was not available in the literature. The density of its hydrous form, Na_2_O·5(B_2_O_3_)·10(H_2_O) (i.e., NaB_5_H_10_O_13_), is known (with ^nat^B, 1.707 g/cm^3^ [48]; with ^10^B, 1.67 g/cm^3^). The density of Na^10^B_5_O_8_ was experimentally measured to be approximately 2.02 g/cm^3^, fixing the density of natural NaB_5_O_8_ at 2.06 g/cm^3^.

The triton from ^6^Li(n,α)^3^H has a range of 62.7 μm, which can easily escape a ~10 µm diameter converter particle to deposit most of its 2.78 MeV energy into the surrounding scintillator material. However, the ^10^B daughters deposit their energy almost an order of magnitude closer to the event origin than the ^3^H from ^6^Li. Thus, the majority of daughter products from a ^10^B(n,α)^7^Li reaction in a 10 µm diameter particle would not escape the particle to interact with any surrounding material, demonstrating the need for smaller boron particles to maximize the probability of conversion from absorbed neutrons in the converter to photon production from surrounding scintillator material.

The neutrons transmitted through a sample carry attenuation information about the sample, which are converted to photons by the scintillator, and read by a digital camera and sampled into pixels to form an image. Thus, the neutron counting statistics dictate image quality as long as the camera detects enough light to represent the number of neutrons absorbed in the scintillator screen. 

Scintillators with a higher detection efficiency can offer shorter acquisition times and improved image quality (e.g., signal-to-noise ratio, SNR). Scintillator screens containing one or more effective converter materials detect neutrons by attenuating them through absorption, so screens that absorb more neutrons demonstrate increased neutron detection efficiency. Neutron attenuation is described by the Beer–Lambert law, shown in Equation (6), where *ϕ*_0_ is the initial neutron flux and *ϕ*(*t*) is the neutron flux after attenuation through a material of thickness, *t*, and total attenuation macroscopic cross-section, *Σ_Total_*. The total attenuation cross-section (*Σ_Total_*) is the sum of the scattering and absorption cross-sections (*Σ_s_* and *Σ_a_,* respectively). For the strongly absorbing materials considered here, *Σ_a_* >> *Σ_s_*, meaning the attenuation is driven by absorption and scattering effects are negligible, and *Σ_Total_* ≈ *Σ_a_*. Thus, a screen’s detection efficiency is the neutron attenuation fraction, (*t*)/*ϕ*_0_:

(6) $$\phi \left(t\right)=\phi_{0}e^{-\Sigma_{total}t}$$

**Figure 3:**
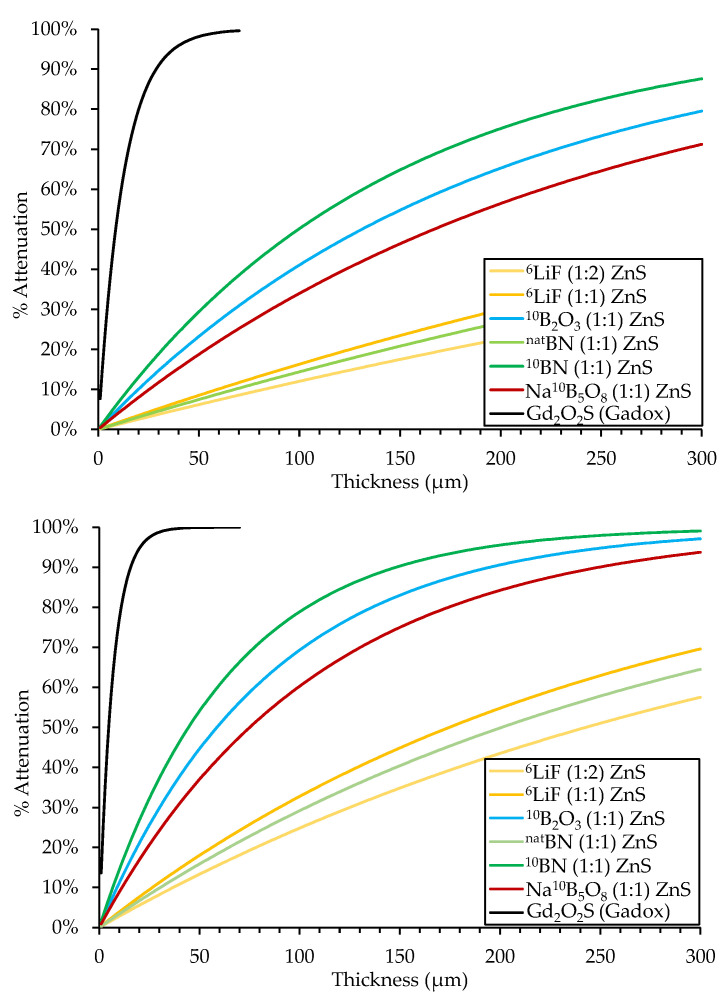
Calculated attenuation fraction for the thermal (**top**) and cold neutrons (**bottom**) in different scintillator materials.

Calculated neutron detection efficiencies of various scintillator materials are listed in Table 2 and displayed in Figure 3. The attenuation curves for both thermal and cold neutrons show that gadolinium-based scintillators have the highest neutron detection efficiency. However, they have a large daughter product energy and are also sensitive to γ-rays. ^10^B-based scintillators have a significantly higher attenuation than ^6^Li scintillators, so a thinner borated scintillator screen can maintain the same detection efficiency as a thicker ^6^Li screen, further improving resolution by minimizing the potential for photon diffusion within the screen. This suggests that ^10^B-based screens should exhibit higher detection efficiency than ^6^Li-based screens, lower γ-ray sensitivity than gadolinium-based screens, and improved spatial resolution compared to both gadolinium-based and ^6^Li-based screens.

## 2. Materials and Methods

This study was divided into two phases, beginning with the initial scoping measurements of a narrow set of screen variations that informed the creation of a broader variety of screens for more detailed measurements. The following sections describe the scintillator screens created as part of this work along with the methods employed to characterize their performance. 

### 2.1. Scintillator Screens

The purpose of this study was to conduct a combinatorial study of boron-based neutron scintillators to determine if (1) borated scintillator screens could offer improved neutron imaging capabilities compared to current widely-used screens and (2) which parameters produced the best performing screens. 

#### 2.1.1. Scintillator Screen Compositions

A total of 25 different boron-based scintillator screens were evaluated as part of the initial scoping studies, which are listed in Table 3. The results of this experiment allowed for a down-selection of scintillator screen parameters that informed the second phase of borated scintillator screen design and evaluation. All screens employed a ZnS:Cu scintillator and had an active area of 50 mm × 50 mm. Four screens layered the scintillator and converter materials separately, while the rest had a mixture of scintillator and converter material particles. These scoping studies included a larger variety of converter materials, and only the top few performing converters were pursued further in subsequent testing.

The second phase of this work examined 50 different boron-based scintillator screens, which are listed in Table 4. At least two of each screen variety were fabricated and tested to reduce any anomalous effects from fabrication. Some additional screens were fabricated with a single thickness for the acquisition of test images. This study utilized a total of 118 borated scintillator screens and six different ^6^LiF screens. Except for a few cases that used a 1.8 mm-thick first-surface mirror (FSM), all screens were mounted on 63 mm × 63 mm square aluminum substrates that were 1 mm thick.

Additional substrate materials were also tested. The active area of scintillator and converter material measured 50 mm × 50 mm and a ZnS:Cu scintillator was used for each screen. The ZnS:Cu scintillator was chosen because cameras used in this study have a higher quantum efficiency in the wavelengths emitted from ZnS:Cu than for ZnS:Ag. Screen performance was measured for sensors with a varying converter material (^10^B_2_O_3_, ^nat^BN, and Na^10^B_5_O_8_), scintillator median particle size (11.5 and 4.7 μm), converter-to-scintillator (boron atom to ZnS molecule) mix ratio (2:1, 1:1, and 1:2), and sensor substrate (FSM, matte black aluminum, polished aluminum, matte finish aluminum, broad-spectrum white polymer-coated aluminum, and broad-spectrum white polymer sheet adhered to aluminum). While the ^nat^BN and Na^10^B_5_O_8_ scintillator materials were obtained commercially, the ^10^B_2_O_3_ was fabricated specifically for this project using a precursor of ^10^B-enriched boric acid (H_3_^10^BO_3_) and basic chemical and material processes.

Three separate types of scintillator screens were tested during this study. First, wedged screens consisting of mixed converter and scintillator material were tested to determine the impact of scintillator layer thickness on imaging performance. Second, screens with separate scintillator and converter layers as wedges of each layer, referred to as double wedge scintillator screens, were fabricated with the two wedges deposited in perpendicular directions so that a single double-wedged screen represents the full range of thicknesses for both the converter and scintillator materials. Third, screens with a thin, single-thickness scintillator layer were used to test some boron-based screens for neutron radiography.

#### 2.1.2. Wedged Scintillator Screens

Screen thickness is one of the most impactful parameters that determines the performance of a scintillator screen. To determine screen performance as a function of thickness, one could fabricate multiple screens with a range of discrete thicknesses, measure the performance of each, and then interpolate between the data points. However, this is expensive and time-consuming. 

**Figure 4:**
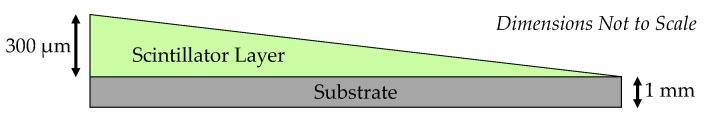
Schematic of a wedged scintillator concept [49].

This work simultaneously studies a continuous range of thicknesses using a wedged scintillator screen, where scintillator layer thickness varies continuously across the screen, as described in previous work [49]. The wedged screens were fabricated by first depositing a nominally 300 μm-thick scintillator layer onto the substrate. Then, a rigid blade was used to scrape the scintillator layer away until the target high and low thicknesses were reached with a monotonic gradient in between them. A jig was used to keep the blade at the proper angle. The resulting screens had a nominal maximum thickness of 300 μm that decreased to a thin layer a few microns thick. The as-fabricated scintillator thickness deposited on the substrate was measured with two different commercial coating thickness gauges that use eddy current probes. The measurements from both gauges were taken at the thicker portion of the screen and averaged together to produce a maximum as-fabricated thickness. A schematic of the wedged scintillator design emphasizing the wedge shape (exaggerated, not to scale) is shown in Figure 4. In total, 84 wedged screens were part of this study. 

**Figure 5:**
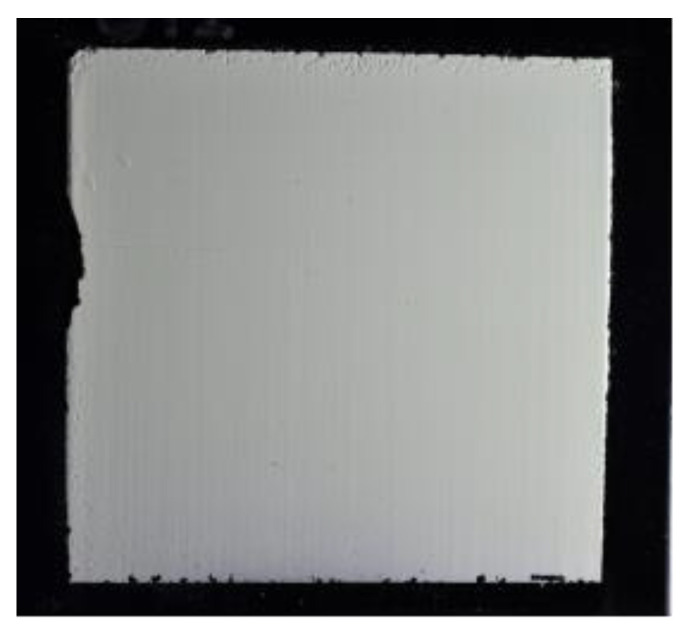
Photograph of a wedged scintillator screen with an oblique light applied to enhance the appearance of the surface texture. The screen shown is a ^10^B_2_O_3_ neutron converter in a 1:1 (^10^B:ZnS) mix ratio with a ZnS:Cu scintillator with 11.5 μm median particle size.

Figure 5 shows a photograph of a representative wedged screen taken with an oblique light applied to enhance the appearance of the surface texture in the picture. Multiple surface defects can be seen in these prototype screens, but these discontinuities are accounted for in the post-processing of experimental data [49]. The thicker section of the wedge is at the top of the image, with the thickness decreasing continuously toward the bottom of the screen.

#### 2.1.3. Double-Wedged Scintillator Screens

**Figure 6:**
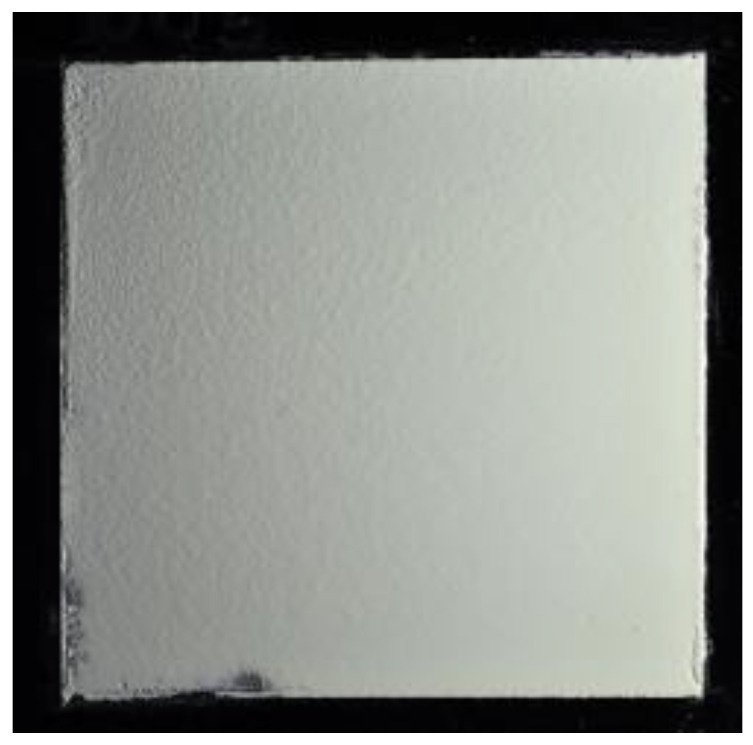
Photograph of a double-wedged scintillator screen consisting of a boron carbide converter (^10^B_4_C) and a ZnS:Cu scintillator with 11.5 μm median particle size.

Mixing the converter and scintillator particles is feasible for white converter particles because they do not absorb the light emitted from the scintillator like a dark-colored material would. This study tested the performance of dark boron-containing converters, including ^10^B_4_C and ^10^B metal, but separated them into a distinct layer from the scintillator in the form of a double-wedged scintillator screen. The converter layer was deposited directly onto an aluminum substrate and the thickness ranged from 100 µm to a few microns. The scintillator layer was deposited on top of the converter layer and varied from 300 μm to a few microns thick. The thickest parts of the two wedges were placed perpendicularly to each other, so each position of the scintillator screen represented a different combination of converter and scintillator layer thickness. This approach allowed a continuous range of thickness combinations to be assessed using a single screen. Nine separate double-wedged screens were included in this study. A double-wedged screen is displayed in Figure 6. The scintillator thickness varies from top (thicker) to bottom (thinner), while the converter is thickest on the right and decreases toward the left of the screen.

#### 2.1.4. High-Resolution Scintillator Screens

**Figure 7:**
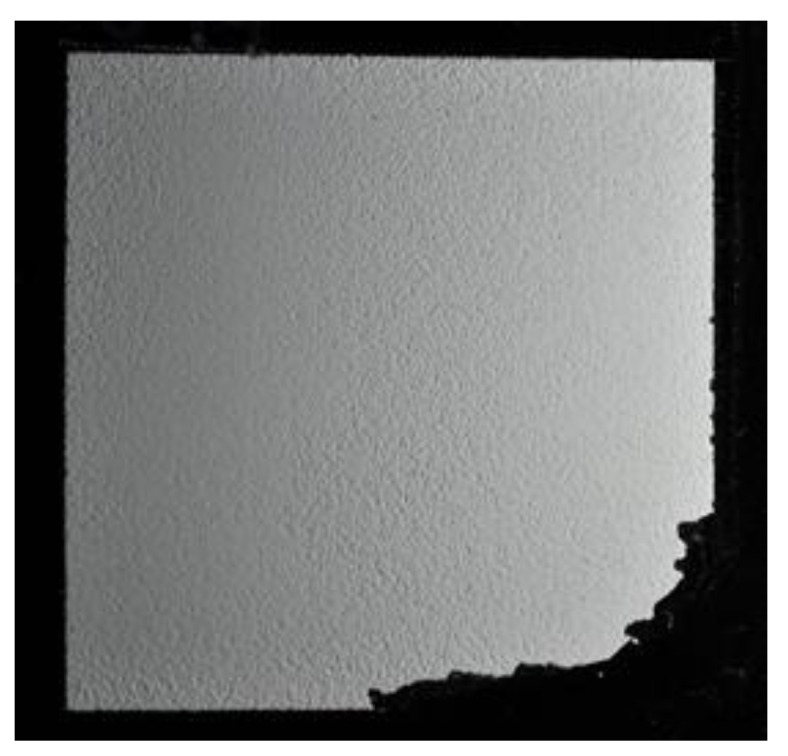
Photograph of a high-resolution scintillator screen consisting of a ^10^B_2_O_3_ neutron converter in a 1:1 (^10^B:ZnS) mix ratio with a ZnS:Cu scintillator with 4.7 μm median particle size.

Boron-based screens with uniformly thick scintillator coatings deposited as thin as possible were also explored in this study. The scintillator layers for these screens all had target maximum thicknesses of ≤ 20 μm. Light dispersion within the scintillator layer is a source of image unsharpness, so thin screens should exhibit higher spatial resolution. This part of the study explored the feasibility of achieving higher spatial resolution by exploiting boron’s increased neutron detection efficiency to offset the lower light output of a thinner screen. Figure 7 displays a photograph of a high-resolution screen under oblique lighting to emphasize screen texture. The missing portion of the coating was caused by the insufficient adhesion of the coating to the substrate during a delicate scraping operation to make the wedged thickness profile. However, this defect did not prohibit measurements because it is outside the central field of view (FOV) of the screen.

### 2.2. Neutron Source and Instrumentation

The measurements for this study were taken using the ANTARES cold neutron beam located at the FRM II reactor [50]. All radiographs were acquired with a neutron flux of 5.7 × 10^7^ n/cm^2^/s at the sample position and a length-to-diameter ratio (*L*/*D*) of 540, where *L* is the beamline length measured from the aperture to the image plane and *D* is the effective diameter of the aperture. A 1 mm-thick cadmium filter was placed in the beamline to filter thermal neutrons for the testing of the screens’ sensitivity to epithermal neutrons. ANTARES has two imaging systems, one with a larger FOV and another for high resolution applications. The large FOV system uses an Andor iKon-L camera (Andor iKon-L, Oxford Instruments, Belfast, UK). The high-resolution imaging system utilizes an Andor Neo camera (Andor Neo, Oxford Instruments, Belfast, UK). The peak light emission of the ZnS:Cu used in this study is ~530 nm, which matches well with the camera used to study light output, as the camera has > 90% quantum efficiency between ~480 and ~710 nm with a peak near 550 nm [51]. The large FOV system was used to simultaneously characterize multiple screens for detection efficiency and light output, and the high-resolution system was used for resolution testing and neutron tomography.

### 2.3. Testing Methodology

The goal of this study was to characterize the performance of a variety of boron-based scintillator screens to understand how different screen parameters affected the final imaging performance. Previous reports of low light output were a concern, so initial scoping studies focused on identifying borated screens that produced sufficient light output for neutron imaging applications and down-selecting the boron converter chemical form to the few most promising candidates for a subsequent set of screens and measurements. The second phase of testing measured the detection efficiency and light yield of the candidate scintillator screens for a wider variety of screen parameters. Spatial resolution was quantified by taking a radiograph of a 1 cm-wide, 75 µm-thick gadolinium strip and calculating the modulation transfer function (MTF) from the edge profile.

**Figure 8:**
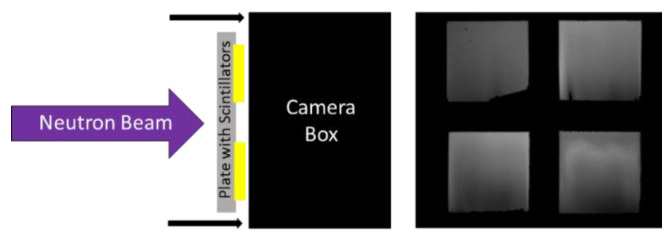
Schematic diagram showing the light yield measurement of the borated screens (**left**). A representative radiograph showing the light output from four borated screens (**right**).

Light output and detection efficiency were measured using previously developed methodology [49]. The larger FOV imaging system was used for these measurements because four screens could be simultaneously measured in a single radiograph. First, radiographs were acquired with the borated screens optically coupled directly to the imaging system to measure the relative light output of each screen. For cold neutron radiographs, this consisted of a single six-second exposure. Epithermal testing was performed by acquiring five 20 s exposures and summing them together. A diagram of this measurement and a sample radiograph from it are displayed in Figure 8.

**Figure 9:**
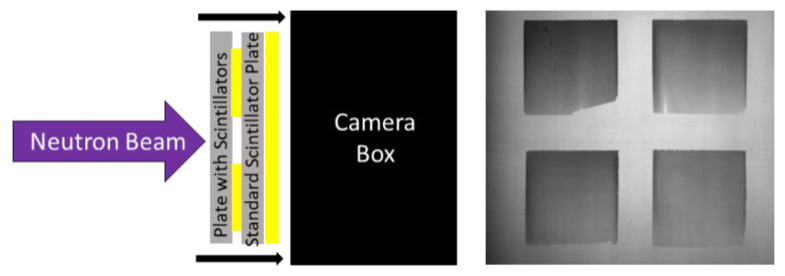
Schematic diagram of the detection efficiency measurement (**left**) and a representative radiograph of four borated screens obtained with this setup (**right**).

Once the radiograph measuring the light yield of the screens was captured, the borated screens were removed from the imaging system and a common ^6^LiF/ZnS:Cu screen was mounted to the imaging system. An open beam image was acquired for the post-process correction of subsequent radiographs. The borated screens were then placed on the source-side of the ^6^LiF/ZnS:Cu screen and a radiograph of the borated screens was acquired to measure the detection efficiency of each screen. Figure 9 shows a schematic of this setup, as well as a representative radiograph of this measurement. There was a gap of only 8 mm between the image plane and the borated screens, so the magnification effects were negligible. This procedure was performed for the double- and single-wedged screens with the cold neutron beam. Epithermal measurements using the same process with a cadmium-filtered beam were also conducted with the single wedge screens. 

### 2.4. Data Processing

A median filter (3 × 3-pixel radius) was applied to all images to reduce noise in the radiographs used to measure relative light output and detection efficiency. All radiographs were open-beam and dark-field corrected. Acquisition times were six seconds for the images taken in the unfiltered cold beam. For the epithermal (i.e., cadmium-filtered) beam, a set of five 20-second images were taken for each measurement and summed together. Spatial resolution images were integrated over ten seconds. A python code was written to correlate data from both the light-yield and detection-efficiency measurements for each screen. Once correlated, relative light yield and detection efficiency as a function of scintillator layer thickness was calculated. A separate python script calculated the effective spatial resolution in terms of MTF. More details on the measurement procedure and data analysis methods can be found in Chuirazzi et al. [49].

## 3. Results

### 3.1. Results of Initial Scoping Studies

**Figure 10:**
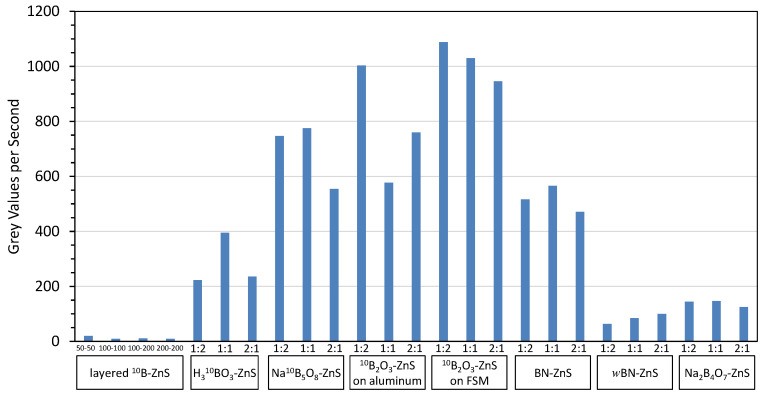
The measured relative light output of borated neutron scintillator screens from the initial scoping studies.

The light outputs of the first set of borated neutron scintillator screens was measured, which are shown in Figure 10. Scintillator screens with layered converter and scintillator material produced minimal light output. The performance of sensors with a converter and scintillator mixture varied based on the converter material and mix ratios. More converter means less scintillator, and vice versa. Too much converter means there is not enough scintillator to produce photons, and too much scintillator means there is not enough converter to absorb the neutrons. There will be an optimal mixture ratio. Thus, the light output does not necessarily change monotonically with the mix ratio.

The highest performing screens in these initial scoping measurements included ^10^B_2_O_3_, ^nat^BN, and Na^10^B_5_O_8_. The light output of the ^10^B_2_O_3_ screens was notably higher for screens on FSM than for a matte black aluminum substrate, indicating that further study of substrate variations may be warranted. Results of this testing informed the fabrication of additional boron-based scintillator screens. This second generation of screens focused on ^10^B_2_O_3_, ^nat^BN, and Na^10^B_5_O_8_ converter materials.

### 3.2. Additional Testing

Converter material, converter grain size, converter-to-scintillator mix ratio, and substrate material were all varied to investigate their impact on the scintillator screens’ performance. The results of the data taken in the cold neutron beam are reported first, followed by epithermal neutron results. Duplicate screens were fabricated with the same combination of screen parameters, and the results of duplicate screens were averaged to reduce the effects of any outliers. Relative light yield is normalized to the single brightest screen to facilitate a relative comparison between all screens.

#### 3.2.1. Cold Neutron Results

**Figure 11:**
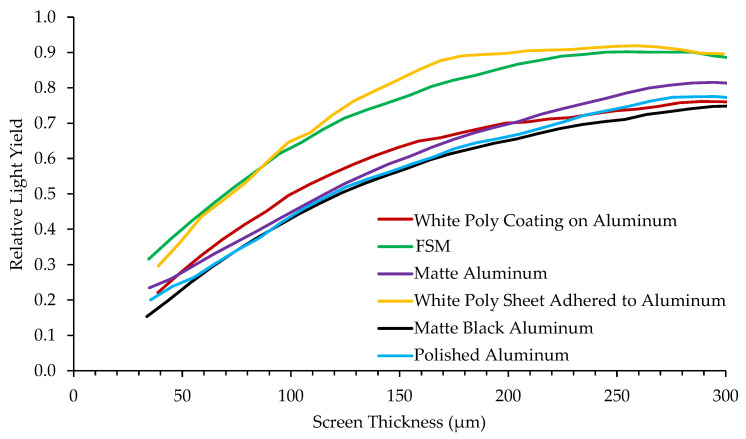
Relative light yield as a function of thickness for a ^10^B_2_O_3_(1:1)ZnS:Cu scintillator composition deposited onto various substrates.

Figure 11 shows relative light yield as a function of screen thickness for the same ^10^B_2_O_3_(1:1)ZnS:Cu scintillator composition deposited on various substrates. Reflective materials such as white poly and a first surface mirror (FSM) increase light output because they simply reflect photons originally traveling away from the camera back toward the camera. However, photons reflected off the substrate undergo further diffusion in the scintillator material with the increased path-length, degrading the spatial resolution. The matte black backing absorbs photons so they do not reflect back and contribute to the image, which reduces light output but can improve spatial resolution. These measurements show that a reflective substrate can increase the relative light yield by > 20% compared to a matte black substrate.

**Figure 12:**
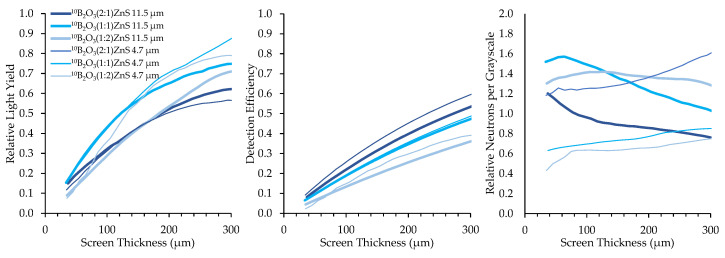
Results for ^10^B_2_O_3_ screens with different mix ratios and scintillator particle sizes.

**Figure 13:**
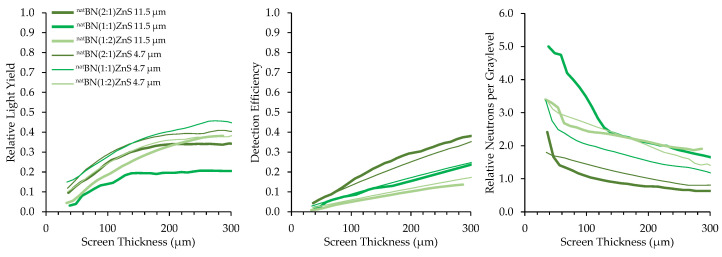
Results for ^nat^BN screens with different mix ratios and scintillator particle sizes.

**Figure 14:**
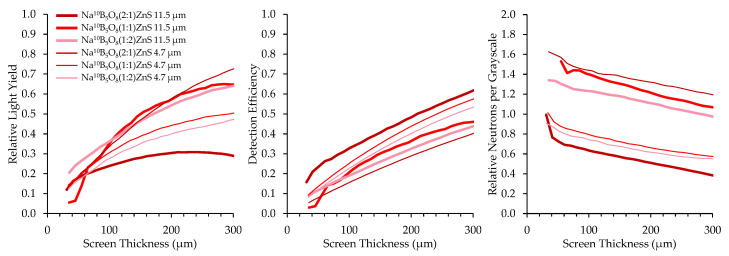
Results for Na^10^B_5_O_8_ screens with different mix ratios and scintillator particle sizes.

Converter materials of ^nat^BN, ^10^B_2_O_3_, and Na^10^B_5_O_8_ were mixed with ZnS:Cu scintillator material in B:ZnS atomic ratios of 2:1, 1:1, and 1:2. Results of borated screens with different converter materials, scintillator particles, and scintillator particle size are displayed in Figure 12, Figure 13 and Figure 14. All screens in these results were mounted on a matte black substrate to remove effects of photon reflection by the substrate. 

Relative neutrons per graylevel, which is the ratio of the detection efficiency to the relative light yield, provides a measure of how many neutrons are represented in a grayscale value and a useful measure to compare screen performance. Detection efficiency scales with the amount of boron converter material, simply due to the increased macroscopic absorption cross-section of the converter–scintillator mixture. However, the screens with higher detection efficiency exhibited fewer photons produced per detected neutron because an increase in the amount of boron decreases the amount of phosphor material, inhibiting photon production. 

Two sizes of scintillator particles, 11.5 and 4.7 μm, were used in the fabrication of the screens. However, there was not a consistent trend in the performance of screens with different sized scintillator particles. Detection efficiency was not affected by scintillator particle size because the bulk boron concentration of the mixture is independent of scintillator particle size. Optical examination did not reveal any differences in homogeneity between screens with different scintillator particle sizes.

**Figure 15:**
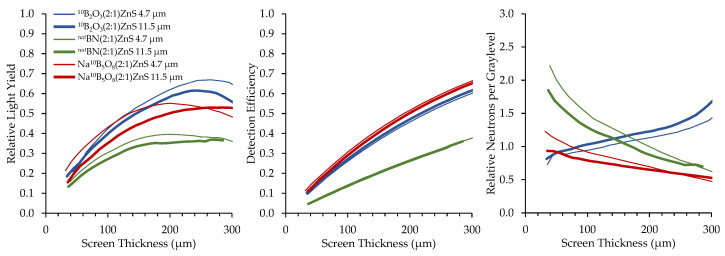
Results for neutron scintillator screens with different neutron converters.

A comparison of the performance of the different neutron converters with a 2:1 mix ratio, mounted to a polished aluminum substrate, is shown in Figure 15. ^10^B_2_O_3_ produced the highest light output while Na^10^B_5_O_8_ provided the best detection efficiency. The ^nat^BN contained unenriched boron, which explains its relatively poor performance compared to the other screens that used enriched ^10^B. However, based solely on the limited amount of ^10^B present in naturally occurring boron (19.9% ^10^B, 80.1% ^11^B), ^10^BN may be superior to ^10^B_2_O_3_ and Na^10^B_5_O_8_ if it were available for future testing.

**Figure 16:**
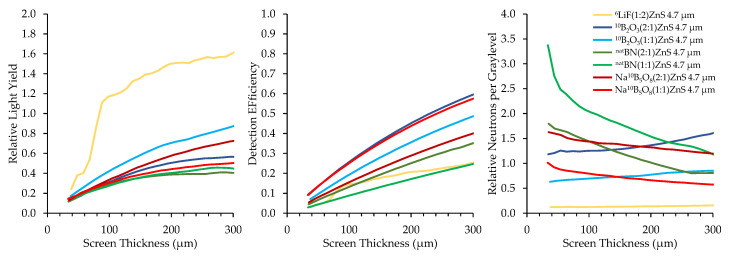
Top-performing boron-based screens under a cold neutron beam compared to a ^6^LiF:ZnS screen.

The top performing boron-based screens were compared with a ^6^LiF/ZnS:Cu screen comprised of a 1:2 (^6^LI:ZnS) mix ratio under cold neutron exposure, for which the results are summarized in Figure 16. All scintillator screens had a matte black backing.

The ^6^LiF-based screens produce significantly higher relative light yield than the boron-based screens, which is to be expected based on the higher reaction energy and longer range of ^6^Li’s daughter products. However, the boron-based screens exhibit a much higher detection efficiency than the ^6^Li-based screen, with some screens demonstrating 125% higher neutron detection efficiency for the same thickness. Their higher detection efficiency enables boron screens to provide improved neutron counting statistics, potentially accelerating image acquisition times proportionally to the improvement in detection efficiency. 

The metric of relative neutrons per graylevel indicates that all boron-based scintillator screens correlate with improved neutron statistics compared to ^6^LiF screens. These measurements show that ^6^LiF screens are brighter, but the brightness is not caused by the detection of additional neutrons. Borated scintillator screens that are thinner than ^6^LiF could offer the same neutron detection efficiency but improved resolution. These results align with theoretical predictions and justify the further exploration of boron-based scintillators in neuron imaging applications.

#### 3.2.2. Epithermal Neutron Results

The absorption cross-section of ^10^B is also higher than that of ^6^Li in the epithermal energy range, suggesting that ^10^B may exhibit superior performance in epithermal neutron imaging applications. Epithermal neutron imaging is useful when examining large, dense samples because epithermal neutrons have greater penetration than thermal neutrons, usually at the expense of contrast [52]. Epithermal neutron imaging is used to examine both archeological artifacts [53,54] as well as nuclear fuels [4,55], among other objects. The epithermal region, which overlaps with the resonance region, contains many absorption resonances for different materials. These resonances can be exploited using pulsed neutrons and time-of-flight techniques to determine the isotopic composition of a material [56]. Neutron scintillator screens with an improved detection efficiency for epithermal neutrons could also be useful in resonance imaging techniques [57,58,59].

**Figure 17:**
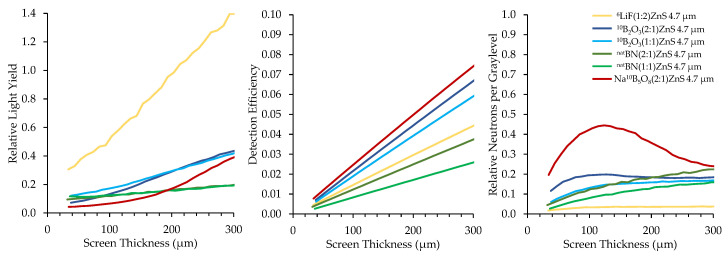
Top-performing boron-based screens under an epithermal neutron beam compared to a ^6^LiF:ZnS screen.

In this work, a 1 mm-thick sheet of cadmium was placed in the neutron beam to absorb thermal neutrons, leaving primarily epithermal neutrons. Borated scintillator screen performance was measured under epithermal neutron exposure following the same procedure as with the unfiltered neutron beam. Figure 17 shows the top performing boron-based scintillators compared to a common ^6^LiF scintillator. The ^6^LiF scintillator exhibited superior light output compared to the borated screens under epithermal neutron exposure, as was the case with cold neutrons. However, the boron-based screens exhibit a much higher detection efficiency than the ^6^Li-based screen, with some screens exhibiting 67% higher epithermal neutron detection efficiency for the same thickness. Epithermal neutron tomography can take more than a day to acquire a full set of projections, but these results show that boron-based screens have the potential to reduce the time necessary for an epithermal neutron tomography by approximately 60%. Additionally, the relative neutrons per graylevel is significantly higher for the boron-based scintillators than for the ^6^LiF scintillator, which could indicate improved SNR for the images produced using the boron-based scintillators. 

#### 3.2.3. Spatial Resolution

**Figure 18:**
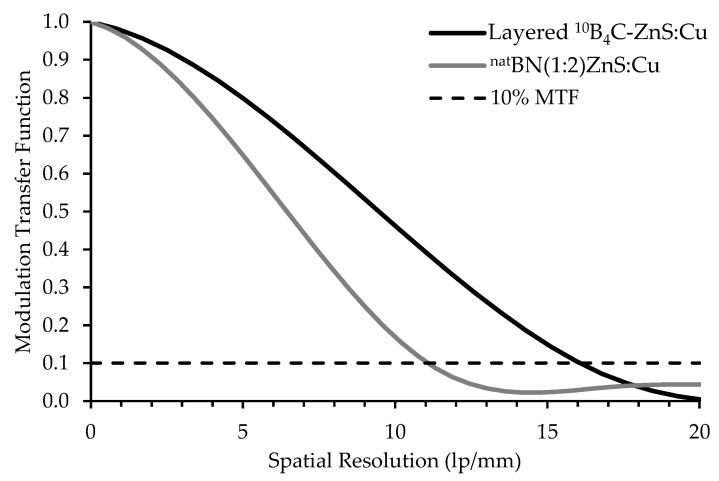
MTF curves of high-resolution scintillators with the best spatial resolution. The boron carbide screen is layered with ZnS, while the boron nitride screen is mixed with the ZnS scintillator.

Spatial resolution of the high-resolution boron screens was measured using the high-resolution imaging system at ANTARES. The effective pixel pitch of this system was 57 pixels/mm (17.5 µm/pixel), corresponding to a Nyquist frequency of 28.5 lp/mm. Figure 18 shows the MTF results for the high-resolution screens, which consisted of thinly deposited (10–20 μm) scintillator layers with 4.7 μm median scintillator particle size. The spatial resolution of all high resolution screens is displayed in Table 5 and the MTF curves of the top two performers, a layered ^10^B_4_C/ZnS screen and a ^nat^BN/ZnS screen, are displayed in Figure 18. Discrepancies in the spatial resolution for screens of the same material can be attributed to variations in the already the thin (≤20 μm) target scintillator layer thickness. At thin scintillator layers, a slight variation in the amount of deposited scintillator material or surface defects can drastically affect screen performance. 

#### 3.2.4. Neutron Computed Tomography

**Figure 19:**
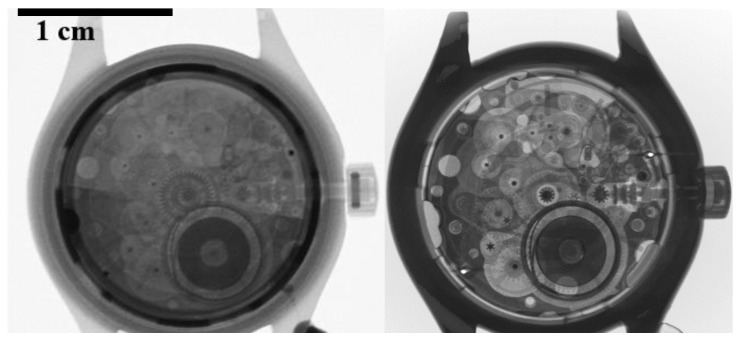
A neutron radiograph (**left**) and a cut-away of a 3D tomographic reconstruction (**right**) of a mechanical wristwatch.

Radiographs were acquired with some of the high-resolution scintillator screens. These screens were chosen based on their promising performance, but also for the relatively uniform surface texture in the central field of view of the screens. Figure 19 (left) shows a radiograph of a small mechanical wristwatch acquired using a thin ^10^B_2_O_3_/ZnS:Cu screen. A set of 1229 radiographs was acquired for neutron computed tomography, and the resulting 3D rendering of the wristwatch is displayed in the right side of Figure 19. The mechanical gears inside the watch are clearly visible, demonstrating that these prototype screens are feasible to use in neutron radiography and tomography applications.

## 4. Discussion

Boron-based scintillator screens demonstrated superior neutron detection efficiency for both cold and epithermal neutron applications when compared to widely used ^6^LiF/ZnS neutron scintillator screens. Borated screens demonstrated up to 125% higher cold neutron detection efficiency and 67% higher epithermal neutron detection efficiency than representative ^6^LiF screens. Although borated screens produce less light output than representative ^6^LiF screens, the borated screens deliver improved neutron counting statistics compared to representative ^6^LiF screens. The higher neutron detection efficiency of boron-based screens offers the potential for higher quality neutron images and lower acquisition times. Epithermal neutron tomography acquisition time can potentially be reduced from days to hours, depending on the application.

Thin boron-based scintillator screens were measured to compare their spatial resolution performance. While some of the boron-based screens provided relatively high-resolution performance, the resolution performance was limited by the Nyquist limit of the imaging system employed in this study. Further measurements should be pursued using a neutron imaging system with a higher Nyquist limit to determine the spatial resolution potential of boron-based screens. 

The top performing screens successfully produced a useful tomographic reconstruction of a test object. Boron-based screens may be able to deliver improved spatial resolution over common widely used screens based on the fundamental properties of the neutron absorption daughter products of ^10^B compared to those of ^6^Li. Provided an imaging setup has sufficient light sensitivity, boron-based neutron scintillators can provide improved neutron detection statistics. The higher neutron detection efficiency of boron-based screens stands to benefit the world-wide neutron imaging community by increasing neutron counting statistics and reducing measurement times. 

## Figures and Tables

**Table 1:** Simulated converter daughter product range in converter medium.

Converter	Density (g/cm^3^)	Daughter Product Range			
		^7^Li (1.01 MeV)	α (1.78 MeV)	^7^Li (0.84 MeV)	α (1.47 MeV)
B_2_O_3_	2.46	2.45 µm	4.84 µm	2.19 µm	3.97 µm
NaB_5_O_8_	2.06	2.98 µm	5.88 µm	2.66 µm	4.82 µm
BN	2.10	2.61 µm	5.21 µm	2.34 µm	4.24 µm
		^3^H (2.73 MeV)	α (2.05 MeV)		
LiF	2.64	62.7 µm	6.04 µm		
		IC_e−_ (29 keV)	IC_e−_ (71 keV)		
^157^Gd_2_O_2_S:Tb	7.32	1.31 µm	5.46 µm		

**Table 2:** Total macroscopic cross-section, mean free path, and the 20% absorption thickness of different borated scintillator screens compared to ^6^LiF and GOS screens.

Scintillator Mixture	Σ_total_		Mean Free Path (µm)		20% Absorption Thickness (µm)	
	(cm^−1^)					
	Thermal	Cold	Thermal	Cold	Thermal	Cold
^6^LiF:ZnS (1:2)	12.8	28.5	780	350	174	78
^nat^BN:ZnS (1:1)	14.3	32.0	700	313	156	70
^6^LiF:ZnS (1:1)	17.8	39.7	562	252	125	56
Na^10^B_5_O_8_:ZnS (1:1)	41.5	92.4	241	108	54	24
^10^B_2_O_3_:ZnS (1:1)	52.9	118	189	85	42	19
^10^BN:ZnS (1:1)	69.6	156	144	64	32	14
Gd_2_O_2_S (Gadox)	799	1467	13	6.8	2.8	1.5

**Table 3:** Parameters of screens used in initial scoping studies.

Substrate	Converter Material	Atomic Ratio (B:ZnS)	Thickness (µm)
Aluminum	^10^B-enriched boron powder	layered	5050
Aluminum	^10^B-enriched boron powder	layered	100,100
Aluminum	^10^B-enriched boron powder	layered	200,100
Aluminum	^10^B-enriched boron powder	layered	200,200
Aluminum	^10^B-enriched Boric Acid	2:1	200
Aluminum	^10^B-enriched Boric Acid	1:1	200
Aluminum	^10^B-enriched Boric Acid	1:2	200
Aluminum	^10^B-enr. Sodium Pentaborate	2:1	200
Aluminum	^10^B-enr. Sodium Pentaborate	1:1	200
Aluminum	^10^B-enr. Sodium Pentaborate	1:2	200
Aluminum	Boron Oxide (B_2_O_3_)	2:1	200
Aluminum	Boron Oxide (B_2_O_3_)	1:1	200
Aluminum	Boron Oxide (B_2_O_3_)	1:2	200
1.9 mm FSM	Boron Oxide (B_2_O_3_)	2:1	200
1.9 mm FSM	Boron Oxide (B_2_O_3_)	1:1	200
1.9 mm FSM	Boron Oxide (B_2_O_3_)	1:2	200
Aluminum	Boron Nitride (BN)	2:1	200
Aluminum	Boron Nitride (BN)	1:1	200
Aluminum	Boron Nitride (BN)	1:2	200
Aluminum	Wurtzite-Boron Nitride (w-BN)	2:1	200
Aluminum	Wurtzite-Boron Nitride (w-BN)	1:1	200
Aluminum	Wurtzite-Boron Nitride (w-BN)	1:2	200
Aluminum	Anhydrous Sodium Tetraborate	2:1	200
Aluminum	Anhydrous Sodium Tetraborate	1:1	200
Aluminum	Anhydrous Sodium Tetraborate	1:2	200

**Table 4:** Scintillator screen parameter combinations of second testing phase.

Particle Size (µm)	Phosphor Maximum Thickness	Converter Material	Converter Thickness (µm)	Converter to Scintillator At. Ratio (B:ZnS)	Converter to Scintillator Weight Ratio	Substrate Surface Finish	Notes
11.5	300	^10^B metal	100	-	-	matte black	double Wedge
4.7	300	^10^B metal	100	-	-	matte black	double Wedge
11.5	300	^10^B_4_C	100	-	-	matte black	double Wedge
4.7	300	^10^B_4_C	100	-	-	matte black	double Wedge
11.5	300	^10^B_2_O_3_	-	2:1	1:2.5	matte black	single wedge
11.5	300	^10^B_2_O_3_	-	1:1	1:4	matte black	single wedge
11.5	300	^10^B_2_O_3_	-	1:2	1:7	matte black	single wedge
4.7	300	^10^B_2_O_3_	-	2:1	1:2.5	matte black	single wedge
4.7	300	^10^B_2_O_3_	-	1:1	1:4	matte black	single wedge
4.7	300	^10^B_2_O_3_	-	1:2	1:7	matte black	single wedge
11.5	300	Na^10^B_5_O_8_	-	2:1	1:2.3	matte black	single wedge
11.5	300	Na^10^B_5_O_8_	-	1:1	1:3.5	matte black	single wedge
11.5	300	Na^10^B_5_O_8_	-	1:2	1:6.1	matte black	single wedge
4.7	300	Na^10^B_5_O_8_	-	2:1	1:2.3	matte black	single wedge
4.7	300	Na^10^B_5_O_8_	-	1:1	1:3.5	matte black	single wedge
4.7	300	Na^10^B_5_O_8_	-	1:2	1:6.1	matte black	single wedge
11.5	300	^nat^BN	-	2:1	1:3.1	matte black	single wedge
11.5	300	^nat^BN	-	1:1	1:5.1	matte black	single wedge
11.5	300	^nat^BN	-	1:2	1:9.2	matte black	single wedge
4.7	300	^nat^BN	-	2:1	1:3.1	matte black	single wedge
4.7	300	^nat^BN	-	1:1	1:5.1	matte black	single wedge
4.7	300	^nat^BN	-	1:2	1:9.2	matte black	single wedge
11.5	300	^10^B_2_O_3_	-	2:1	1:2.5	polished	single wedge
11.5	300	^10^B_2_O_3_	-	1:1	1:4	polished	single wedge
11.5	300	^10^B_2_O_3_	-	1:2	1:7	polished	single wedge
4.7	300	^10^B_2_O_3_	-	2:1	1:2.5	polished	single wedge
4.7	300	^10^B_2_O_3_	-	1:1	1:4	polished	single wedge
4.7	300	^10^B_2_O_3_	-	1:2	1:7	polished	single wedge
11.5	300	Na^10^B_5_O_8_	-	2:1	1:2.3	polished	single wedge
11.5	300	Na^10^B_5_O_8_	-	1:1	1:3.5	polished	single wedge
11.5	300	Na^10^B_5_O_8_	-	1:2	1:6.1	polished	single wedge
4.7	300	Na^10^B_5_O_8_	-	2:1	1:2.3	polished	single wedge
4.7	300	Na^10^B_5_O_8_	-	1:1	1:3.5	polished	single wedge
4.7	300	Na^10^B_5_O_8_	-	1:2	1:6.1	polished	single wedge
11.5	300	^nat^BN	-	2:1	1:3.1	polished	single wedge
11.5	300	^nat^BN	-	1:1	1:5.1	polished	single wedge
11.5	300	^nat^BN	-	1:2	1:9.21	polished	single wedge
4.7	300	^nat^BN	-	2:1	1:3.1	polished	single wedge
4.7	300	^nat^BN	-	1:1	1:5.1	polished	single wedge
4.7	300	^nat^BN	-	1:2	1:9.2	polished	single wedge
11.5	300	^10^B_2_O_3_	-	1:1	1:4	white poly coating	single wedge
11.5	300	^10^B_2_O_3_	-	1:1	1:4	FSM	single wedge
11.5	300	^10^B_2_O_3_	-	1:1	1:4	matte	single wedge
11.5	300	^6^LiF	-	1:2	1:9.2	matte black	single wedge
6.7	300	^6^LiF	-	1:2	1:9.2	matte black	single wedge
4.7	300	^6^LiF	-	1:2	1:9.2	matte black	single wedge
11.5	300	^6^LiF	-	1:2	1:9.2	matte	single wedge
6.7	300	^6^LiF	-	1:2	1:9.2	matte	single wedge
4.7	300	^6^LiF	-	1:2	1:9.2	matte	single wedge
11.5	300	^10^B_2_O_3_	-	1:1	1:4	white poly sheet	single wedge
4.7	20	^10^B_2_O_3_	-	2:1	1:2.5	matte black	
4.7	20	^10^B_2_O_3_	-	1:1	1:4	matte black	
4.7	20	^10^B_2_O_3_	-	1:2	1:7	matte black	
4.7	20	Na^10^B_5_O_8_	-	2:1	1:2.3	matte black	
4.7	20	Na^10^B_5_O_8_	-	1:1	1:3.5	matte black	
4.7	20	Na^10^B_5_O_8_	-	1:2	1:6.1	matte black	
4.7	20	^nat^BN	-	2:1	1:5.7	matte black	
4.7	20	^nat^BN	-	1:1	1:10.4	matte black	
4.7	20	^nat^BN	-	1:2	1:19.9	matte black	
4.7	20	pure ^10^B	10	-	-	matte black	layered
4.7	20	^10^B_4_C	10	-	-	matte black	layered

**Table 5:** Effective spatial resolution for uniform thickness screens.

Converter Material	Converter to Scintillator Atomic Ratio (Boron atom to ZnS:Cu)	Resolution at 10% Modulation Transfer Function (MTF) (lp/mm)
^10^B_2_O_3_	2:1	10.6
^10^B_2_O_3_	2:1	7.6
^10^B_2_O_3_	1:1	9.6
^10^B_2_O_3_	1:1	9.6
^10^B_2_O_3_	1:2	7.4
^10^B_2_O_3_	1:2	7.3
^10^NaB_5_O_8_	2:1	10.5
^10^NaB_5_O_8_	2:1	8.8
^10^NaB_5_O_8_	1:1	10.2
^10^NaB_5_O_8_	1:1	9.9
^10^NaB_5_O_8_	1:2	7.6
^10^NaB_5_O_8_	1:2	8.2
^nat^BN	2:1	4.8
^nat^BN	2:1	9.7
^nat^BN	1:1	8.3
^nat^BN	1:1	6.7
^nat^BN	1:2	6.6
^nat^BN	1:2	15.2
Layered ^10^B_4_C	10 μm ^10^B_4_C, 20 μm ZnS:Cu	16.0
